# Wireless electrochemical actuation of soft materials towards chiral stimuli

**DOI:** 10.1039/d2cc06630k

**Published:** 2023-01-25

**Authors:** Serena Arnaboldi

**Affiliations:** a Università degli Studi di Milano, Dipartimento di Chimica Via Golgi 19 20133 Milano Italy serena.arnaboldi@unimi.it

## Abstract

Different areas of modern chemistry, require wireless systems able to transfer chirality from the molecular to the macroscopic event. The ability to recognize the enantiomers of a chiral analyte is highly desired, since in the majority of cases such molecules present different physico-chemical properties that could lead, eventually, to dangerous or harmful interactions with the environment or the human body. From an electrochemical point of view, enantiomers have the same electrochemical behavior except when they interact in a chiral environment. In this Feature Article, different approaches for the electrochemical recognition of chiral information based on the actuation of conducting polymers are described. Such a dynamic behavior of π-conjugated materials is based on an electrochemically induced shrinking/swelling transition of the polymeric matrix. Since all the systems, described so far in the literature, are achiral and require a direct connection to a power supply, new strategies will be presented in the manuscript, concerning the implementation of chirality in electrochemical actuators and their use in a wireless manner through bipolar electrochemistry. Herein, the synergy between the wireless unconventional actuation and the outstanding enantiorecognition of inherent chiral oligomers is presented as an easy and straightforward read out of chiral information in solution. This approach presents different advantages in comparison to classic electrochemical systems such as its wireless nature and the possible real-time data acquisition.

## Abstract 2

The synergy between wireless unconventional actuation and chiral stimuli can be used as a straightforward read out of chiral information.

## Graphical abstract

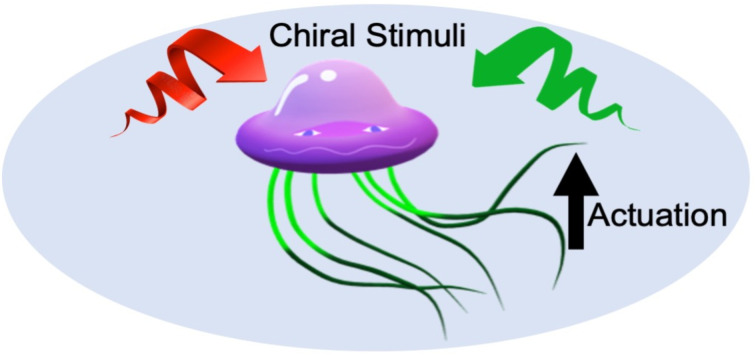

## Introduction

In late 90s, a smart material was defined as a system that responds in a controllable and reversible way to external stimuli.^1^ In such a category physico-chemically driven actuators have the ability to change the shape, stiffness, position, natural frequency and/or other mechanical characteristics in response to changes in temperature and electric or magnetic fields. Different types of solid state actuators have been reported, including shape memory alloys, piezoelectrics, magnetostrictive or electrostrictive devices and electroactive polymers (EAPs).^2^ The latter, have gained considerable attention due to their large deformation transitions, biocompatibility and processability. Polymeric materials have been exploited in several fields of research, where lightweight, flexible and high energy efficiency materials are required. Commonly electroactive polymers can be categorized into two main groups: dielectric and ionic EAPs. Dielectric EAPs include dielectric elastomers, ferroelectric polymers, electrostrictive graft elastomers, and liquid crystal elastomers. On the other hand, ionic EAPs can be divided into ionic polymer gels, conductive polymers (CPs), and ionic polymer–metallic composites.^2–10^ Among these soft materials, CPs stand out due to their outstanding electric, optical and mechanical properties.^11^ In this frame, CPs appear particularly attractive as chassis materials to design soft actuators since their conformational changes result from an electrochemical charging/discharging process. The electromechanical properties of several CPs, such as polyanilines, polypyrroles, and polythiophenes have been described in order to design efficient actuating systems.^12–15^ Nonetheless, the majority of the reported systems require a direct electrical connection in order to trigger the dynamic event, which limits its use as an optical readout of chemical information in solution. Thus, the possibility to combine the electromechanical response to far more complex physico-chemical properties such as chirality is a fascinating challenge. Furthermore, the ability of wirelessly triggering mechanical deformation in order to develop easy and straightforward read-outs of chiral information, opens up novel and interesting research lines, especially having in mind the increasing popularity of smartphones combined with a portable potentiostat.^16,17^ For example, recently the wireless concept of bipolar electrochemistry was coupled with chirality to develop new unconventional electrochemical systems that are able to transfer the chiral stimuli from the molecular level to the macroscopic scale through 2D and 3D dynamic approaches.^18^ In this Feature Article, at first, the basic concepts of electromechanical actuation of π-conjugated polymers are presented, followed by a description of the bipolar electrochemistry approach and its possible use to trigger actuation. Afterwards the outstanding synergy between wireless electrochemistry and chiral recognition is illustrated.

### Electromechanical actuation of conducting polymers

**Fig. 1:**
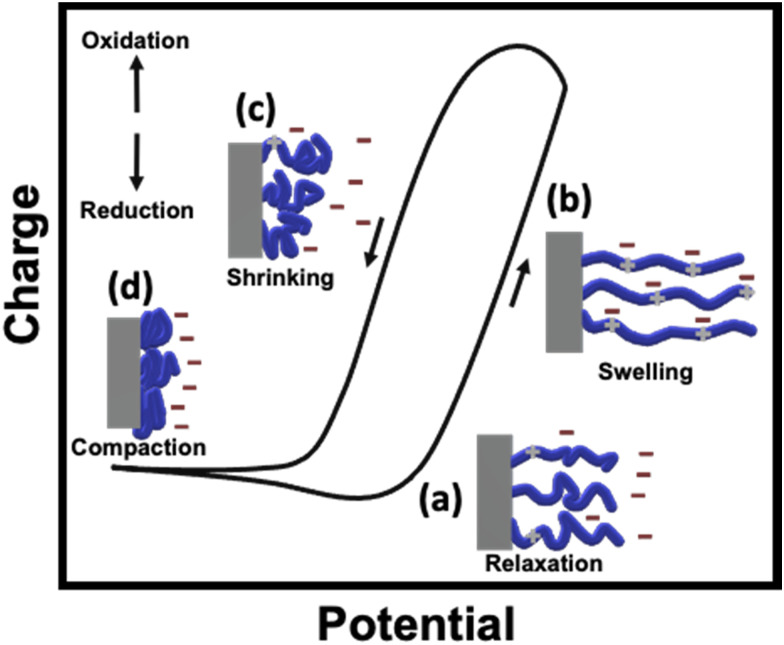
Characteristic coulovoltammogram obtained during the charging/discharging process of a neutral and partially de-doped polymer with a schematic representation of the different conformational changes within the polymeric matrix: (a) relaxation, (b) swelling, (c) shrinking and (d) compaction.

As stated above, conducting polymers are one of the most interesting materials for designing soft-matter actuators, due to their outstanding flexibility, high strength and conductivity. CP-based actuators have been designed with different shapes such as films, filaments and textiles.^19–21^ Electromechanical actuation of these materials is based on the uptake/release of ions associated with the charging/discharging transition of the conjugated backbone of CPs. According to the electrochemically stimulated conformational relaxation model (ESCR), such a process leads to four conformational changes within the polymeric matrix: swelling, shrinking, compaction and relaxation.^22^ Otero's group has studied such conformational changes by evaluating the coulovoltammetric and dynamovoltammetric response during the actuation of Ppy strips in a classic three electrode system.^22^ Commonly, such a shrinking/swelling transition is intimately related to the initial redox state and composition of the polymer matrix. For example, for a neutral and partially de-doped polymer, at first, the initial charging causes a slow uptake of anions (relaxation process) in order to compensate the positive charges formed within the polymer (Fig. 1a). Afterwards, when a high enough anodic potential is applied, the complete doping of the polymer takes place with the concomitant uptake of anions, causing the elongation of the material (Fig. 1b). On the contrary, when the discharge of the polymer begins, a fast release of anions occurs, leading to the contraction of the film (Fig. 1c). After this point, the compaction process takes place, due to applied cathodic potential (Fig. 1d). Thus, during the electrochemical charging/discharging process, π-conjugated polymers undergo length changes, typically associated with notable variations in elastic moduli, electrical conductivity and electromagnetic absorption spectra. All of these variations can be reflected in mechanical deformations.

The electrochemical stimulation of EAPs involves a switching between their oxidized and reduced states. During such a redox transition, electrons are exchanged from and towards the CPs. The overall electroneutrality is maintained by the uptake and release of ions, supplied by the electrolyte. When a π-conjugated material is polymerized in its oxidized state, the positive charges within the polymer matrix are compensated by the anions. The nature of these anions strongly affects the material properties. For example, if the anions are relatively small, an anionic exchange takes place during the reduction/oxidation transition of the polymer. However, when using bulky anions, negative charges are trapped within the polymer matrix. In this case, in order to keep electroneutrality, a cationic exchange occurs during the redox processes.^22^

**Fig. 2:**
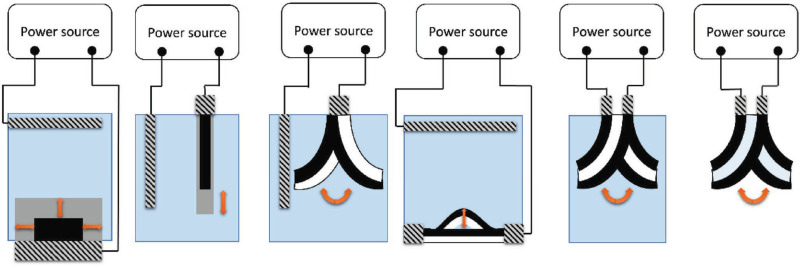
Electrochemical actuation using CPs in different configurations. From left to right; bulk expansion, linear, bending bilayer, buckling sheet, bending trilayer, and bending trilayer in air (Copyright © 2019 WILEY-VCH Verlag GmbH & Co. KGaA, Weinheim).

Among the different CPs, polypyrrole stands out due to its low degree of crystallinity, in comparison with polyaniline, polythiophene and poly-3,4-alkoxythiophenes. In addition, PPy can be easily obtained either by chemical or electrochemical polymerization, in organic or aqueous media, producing free-standing films with relatively highly conductivity. Ppy actuation can be easily achieved with a conventional electrochemical setup; where a Ppy strip is wired to a power supply and used as working electrode. Under such conditions, different electromechanical deformations can be generated, according to the assumed Ppy configuration, *i.e.* bulk, linear, bending bilayer, buckling and bending tri-layer (Fig. 2). However, it is important to highlight that in the following examples, in order to trigger actuation, the symmetry of the system needs to be broken.

Bulk actuators are based on an isotropic volume change, thus the uptake/release of ions takes place simultaneously on all the exposed surfaces of the conducting polymer. For example, Yamada *et al.*^23^ developed a solid-state micro-actuator, where the change in the redox state of the Ppy films, covered with polyethylene glycol in a comb-shaped microelectrode array, produced a simultaneous isotropic shrinking/swelling process. Linear deformation can be achieved by using free-standing films, strips, or fibers, where the ion exchange takes place exclusively on the tip of the film. Although Ppy presents large strain in air but not in solution, different approaches have been proposed to overcome such a limitation by using, (i) ionic liquids as solvents,^24^ (ii) hollow fibers^25^ or (iii) corrugated freestanding PPy films.^26^ However, even with the most promising approach and under well-defined experimental conditions the strain reaches a percentage up to 30%.^27^ Conventional bending of CPs can be achieved by designing a bilayer system in order to break the symmetry of the device. Thus, the change in volume, triggered by the oxidation (or reduction) of the CP layer, generates a stress gradient at the interface CP/passive layer, causing bending of the actuator. Finally, the tri-layer actuators are constituted by a passive layer sandwiched by two CP films. In such a set-up one conducting layer is connected to the working electrode whereas the second one is short-circuited with the reference and counter electrodes.^28^

Although these systems have gained considerable attention, the necessity of a direct electric connection is one of their main constrains. In this context, the wireless configuration presents many advantages, in comparison with the conventional approaches, like the mobility of the device and the possibility of obtaining real-time data acquisition and application directly *in situ*. Furthermore, since the bipolar electrochemistry method allows the asymmetric polarization of a conducting object, it is possible to couple the electromechanical actuation with a redox reaction of interest. Thus, in the following section the concepts of bipolar electrochemistry and its possible use as an external stimuli for electromechanical actuation are described.

### Bipolar electrochemistry for wireless actuation of conducting polymers

**Fig. 3:**
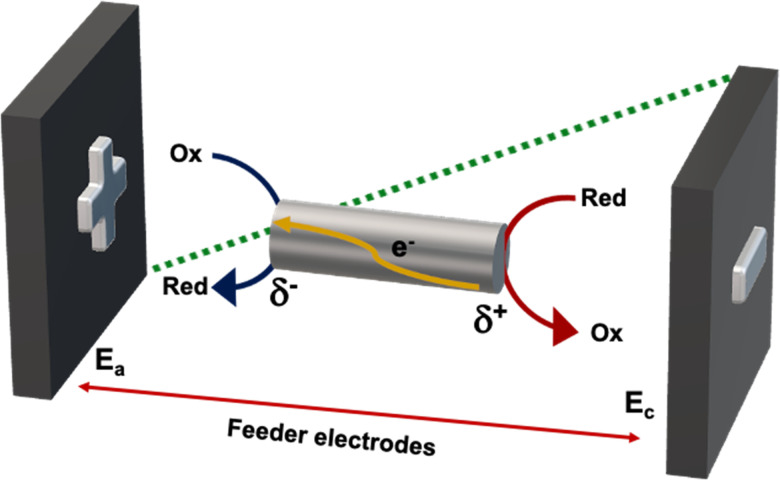
General principle of bipolar electrochemistry.

In recent years bipolar electrochemistry (BE) has been successfully used as a wireless approach in different fields ranging from electrochemiluminescence to electrodissolution and organic synthesis.^29–39^ This method is based on the asymmetric induction of electroactivity on a conducting object or bipolar electrode (BPE), in the presence of an external electric field (*ε*). Under these conditions a potential difference (Δ*V*) between the extremities of the BPE is produced (Fig. 3).

When such a Δ*V* overcomes the thermodynamic threshold potential (Δ*V*_min_), in the presence of electroactive species in solution, redox reactions take place at both sides of the object. Thus, in a first order approximation, the faradaic current that passes through the BPE, from the anodic to the cathodic side, is proportional to the concentration of the analytes under study. Hence the electrochemical activity of a molecule can be coupled with different physico-chemical read outs, *i.e.* optical changes or light emission.^39–42^ Among them, electromechanical actuation is an interesting, easy and straightforward alternative for the optical visualization of chemical information.^43^

However, it is important to take into account different experimental factors before carrying out BE measurements. Briefly, the polarization potential difference generated at the extremities of an anisotropic conducting object, placed orthogonal to the feeder electrodes is given by eqn (1):

1 Δ*V* = *ε l*

where *l* is the length of the bipolar electrode.

Under these conditions the *ε* required to polarize the BPE is a function of the potential difference (Δ*E*) applied between two feeder electrodes separated at a certain distance (*L*) (eqn (2)).

2 *ε* = Δ*E*/*L*

As it can be seen for a given set of *L* and *l* the polarization potential is intimately related to the applied Δ*E*. However, as stated above, a thermodynamic threshold potential (Δ*V*_min_) needs to be overcome in order to trigger the redox reactions. Such a theoretical potential value can be easily estimated by the difference in standard potentials of the redox reactions of interest.

In this frame, the wireless electromechanical deformation of conducting polymers, by imposing an electric field, allows the polarization of the device with respect to the surrounding solution, leading to *δ*^+^ and *δ*^−^ extremities (Fig. 3).

Thus, according to the initial redox state and composition of the polymer matrix, it is possible to couple the oxidation (doping) or overoxidation of the material with its correspondent reduction (de-doping).

**Fig. 4:**
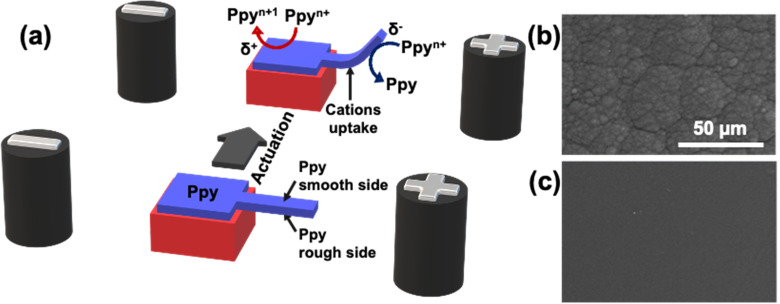
(a) Schematic illustration of the electrochemical actuation mechanism of a Ppy polymer (blue) placed between two feeder electrodes, before and after applying an electric field. The Ppy film is intrinsically asymmetric, having a rough face (b) and a smooth one (c). (b and c) SEM micrographs at 50 μm of the two Ppy sides.

For example, Kuhn *et al.* proposed a mechanism of actuation of polypyrrole based on a double break of symmetry.^44^ In this case a charged polypyrrole doped with dodecylbenzene sulfonate was used as a polymeric actuator. The first break of symmetry is based on the wireless asymmetric polarization of the polymeric cantilever triggered by bipolar electrochemistry. Under these conditions by applying a high enough *ε*, at the *δ*^+^ side, the overoxidation of Ppy occurs, accompanied by a release of cations and a consequent shrinking of the polymer matrix. At the *δ*^−^ extremity, instead, the Ppy reduction is accompanied by an uptake of cations causing a considerable swelling of the polymer (Fig. 4a). The second break of symmetry is based on the intrinsic anisotropic surface roughness of the electrochemically obtained polypyrrole film. In this work, the synthesized Ppy presents a smooth and a rough face which impact the kinetics of the uptake/release of ions (Fig. 4b and c). Briefly, due to the relatively high porosity of the rough face, a fast ion exchange takes place, whereas, the compact structure of the smooth face limits the ion mobility. The combination of such asymmetric uptake/release of ions causes a directional overall bending. It is important to highlight that the relative kinetics of bending are intimately connected to the size of the ion, thus, small ions facilitate the uptake/release transition.

Therefore, the reversibility of the Ppy swelling/shrinking process can be easily fine-tuned by the proper selection of the dopant ion or the applied electric field. For the approaches herein described the dopant used was the anion of the sodium dodecylbenzenesulphonate salt. Thus, the actuation of the synthetized Ppy follows a cation driven mechanism. Such a concept has been used to design electrochemically driven crawlers, pumps, switches, and mechanical transducers of chemical information in solution.^44–50^

In this context, wireless electromechanical actuation was explored for the transduction of chiral information in solution. For example, Assavapanumat *et al.* designed the first wireless enantioselective actuator, by using an imprinted hybrid platinum-Ppy device.^51^ However, in this system, the enantiorecognition is based on the relative kinetic differences which limits its use for the simultaneous analysis of both enantiomers in solution. Thus, in the following section the synergy between inherently chiral recognition and bipolar electrochemistry is presented as an outstanding alternative for the transduction of chiral information.

### Hybrid actuators endowed with chirality in bipolar electrochemistry

**Fig. 5:**
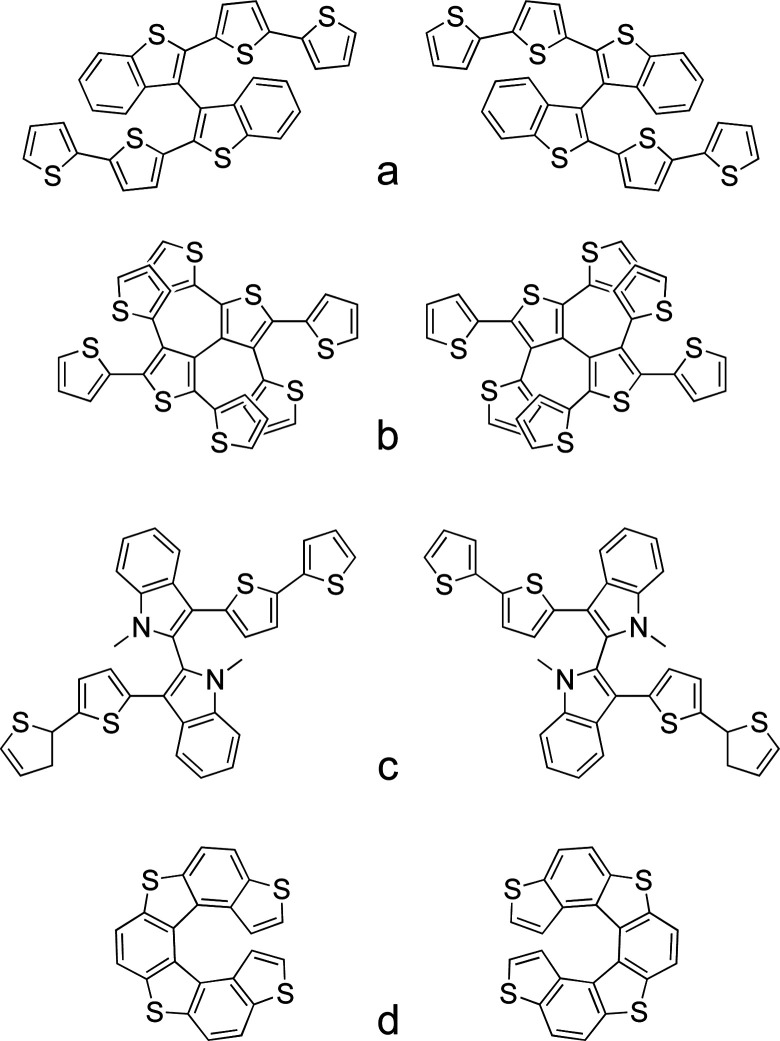
Chemical structures of some of the antipodes of the inherently chiral monomers already studied.

A missing point in the strategies proposed so far in the literature, concerning the design of electrochemical actuators, is the development of chiral systems that are able to transfer chirality across length scales transforming the induced event at the molecular scale, in a triggered dynamic response at the macroscopic level. In this context electroactive heterocycle-based oligomeric films endowed with “inherent” chirality have been recently and successfully used in enantioselective electroanalysis experiments.^52^ In inherently chiral molecular materials, the chirality and their functional properties originate from the same structural element on account of the presence of atropisomeric^53,54^ (Fig. 5a–c) or helicoidal elements^55^ (Fig. 5d) in the main conjugated backbone.

The energy barrier of these materials is too high to be overcome in the working operating conditions, which allows the separation of the racemic mixture into the two stable enantiomers by chiral HPLC. Since all of these molecules have two α-homotopic positions on the terminal thiophenes suitable for electrochemical oligomerization, it is possible to deposit the racemate and enantiopure films on an electrode surface by applying the proper voltage in a classic electrochemical experiment.

These inherently chiral enantiopure electrodes resulted in an exceptionally large peak potential difference (often in the order of 200–300 mV) for the enantiomers of many chiral probes. Importantly, such an impressive enantiodiscrimination ability seems to be a general characteristic, being observed with chiral probes that are quite different in structure and reactivity (*e.g.* 3,4-dihydroxyphenylalanine, ofloxacin and naproxen); by using surfaces prepared from different inherently chiral monomers, in alternative media, and on various electrode supports, including screen printed electrodes. Moreover, due to the neat enantiomer peak separation, such systems allow an estimation of enantiomeric excess.^56^ Since the electron transfer is occurring in a chiral environment (the modified electrode surface) the two redox processes take place at two different thermodynamic potentials, related to the matching/mismatching interactions between the chiral probe and the chiral surface. This potential difference, as it will be demonstrated in the following paragraphs, is crucial for the development of unconventional electrochemical approaches involving chirality and bipolar electrochemistry. In such a context, the synergy between the bipolar electrochemistry and the high stereospecificity of inherently chiral materials plays a pivotal role for the design of enantioselective soft actuators, presenting different advantages; (i) good mechanical properties, due to the presence of a conductive polymer (Ppy), (ii) high enantioselectivity of inherently chiral materials used to functionalize the Ppy chassis, (iii) a wireless-like mode, and as a matter of fact in bipolar electrochemistry the actuator does not require a connection to a power supply.

In particular, the majority of the unconventional electrochemical approaches have been developed involving the deposition of the enantiomers of the monomer nicknamed BT_2_T_4_ (Fig. 5a) on the Ppy chassis.

This molecule presents dibenzothiophene as a central scaffold and it is constituted by two equal moieties, each one being approximately planar and of high effective conjugation. The two α-homotopic positions on the thiophene terminal rings allow its chemical or electrochemical oligomerization.^56^ Herein the Ppy and oligo-BT_2_T_4_ were deposited galvanostatically in a sequential process; first the Ppy chassis was deposited on the surface of a gold slice, followed by the oligomerization of the inherently chiral monomer on the polymeric surface.

Afterwards, the hybrid actuator was easily peeled off and was properly cut in order to design the cantilever-shaped BPE. In addition, to obtain mechanical readouts triggered by chiral stimuli, the Ppy was functionalized just on one side with the enantiopure inherently chiral oligomer.

On the contrary, on the opposite extremity of the BPE, a pristine Ppy polymer was modelled to obtain the cantilever.

Then the hybrid actuator was placed between two feeder electrodes, in a bipolar cell, containing a solution with the chiral probe under study. The final goal was to demonstrate that throughout this system it was possible to absolutely recognize with an on-off approach the enantiomers of a chiral analyte. 3,4-Dihydroxyphenylalanine (DOPA) was chosen as an applicative probe for several reasons; the l-enantiomer of DOPA is synthetized from l-tyrosine by the enzyme tyrosine hydroxylase being the precursor of important catecholamine neurotransmitters such as dopamine, norepinephrine, epinephrine; and eventually it can be used in the treatment of Parkinson's disease. Potentiodynamic measurements carried out in a solution containing l- or d-DOPA, on a free standing Ppy strip functionalized with the (*S*)- or (*R*)-BT_2_T_4_, have demonstrated that the separation between DOPA enantiomers can be of hundreds of mV.

As stated above, this potential difference value is the main ingredient that allows the successful use of bipolar electrochemistry in enantiorecognition applications. Thus, it is possible to transform exclusively l-DOPA when Ppy is functionalized with the (*S*)-oligomer and *vice versa*, d-DOPA with the oligomer in the opposite configuration. The proposed system involves three types of symmetry breaking; (i) intrinsic asymmetry of the Ppy surface roughness; (ii) intrinsic chirality of the oligomer that guarantees separation in terms of peak potential values; (iii) intrinsic asymmetry of bipolar electrochemistry that allows simultaneously two different redox reactions at the bipolar electrode to occur. In particular, a selective oxidation occurs on the extremity where the chiral oligomer is deposited, whereas the reduction of the charged Ppy takes place on the opposite side.^57^

Considering an electrochemical experiment in which an enantiopure electrode surface is dipped in a solution containing two antipodes of a chiral electroactive species, it could be possible to selectively activate just one of the two enantiomers of the chiral analyte under study, by applying a specific electric field value. This feature allows the determination of more than one species when they are both present in solution, since their oxidation occurs at different thermodynamic potential values. It is important to highlight that with alternative enantioselective surfaces, *i.e.* cyclodextrins, metal–organic frameworks or molecular imprinted modified electrodes, such outstanding detection is not possible since in such approaches the differentiation between enantiomers of chiral analytes is in terms of current intensity, without differences in thermodynamic potential values.^18,56,58–78^

### Chiral cantilevers for single detection

**Fig. 6:**
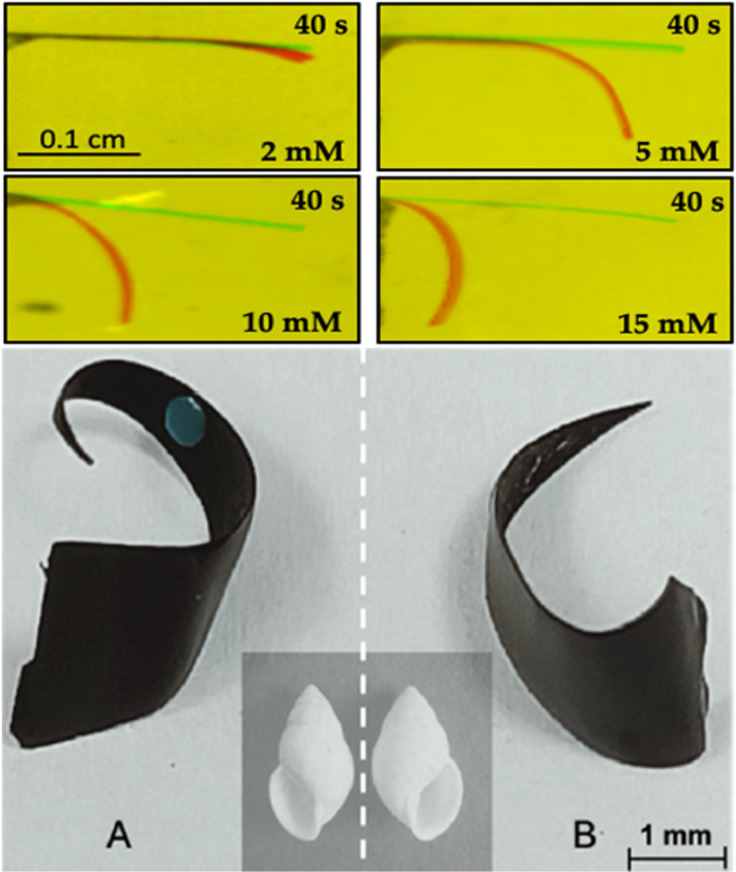
Top: concentration dependent deflection of Ppy for the enantioselective recognition of l-DOPA on oligo-(*S*)-BT_2_T_4_ hybrid actuator (Copyright © 2020, American Chemical Society). Bottom: picture of the two (*R*)- and (*S*)-enantiomorphs recovered at the end of the bipolar experiments illustrating the transmission of molecular chirality to the macroscopic scale (Copyright © 2020, American Chemical Society).

In the first set-up developed the Ppy strip was functionalized on the rough side with the enantiomers of the BT_2_T_4_ oligomer. Bipolar enantiorecognition measurements were carried out towards l- and d-DOPA, by applying a proper electric field. Such systems allow the absolute recognition of, with an on-off approach, the DOPA antipodes; in fact, the mechanical deformation of the Ppy strip takes place just in the case of matching interactions between the oligomer and the analyte in solution (in particular (*S*)-oligo with l-DOPA and (*R*)-oligo with d-DOPA) (Fig. 6 top). The downwards Ppy deformation is related to the ion uptake occurring on the rough side of the strip, causing a swelling of the polymer.^49^ Furthermore the bending degree of these hybrid actuators is concentration dependent, which leads to their possible use as an electroanalytical tool for the quantification of chiral molecules in solution.^57^

The same approach was exploited to obtain enantiomorphic macro-hybrid actuators, where the enantiospecific curling was associated to the matching and mismatching interactions between the object and DOPA antipodes in solution (Fig. 6 on the bottom).^79^ In this case, a hybrid sandwich BPE, composed by Ppy and both (*S*)- and (*R*)-oligo-BT_2_T_4_, was exposed to a proper electric field in the presence of either l- or d-DOPA.

### Chiral cantilevers for double detection

**Fig. 7:**
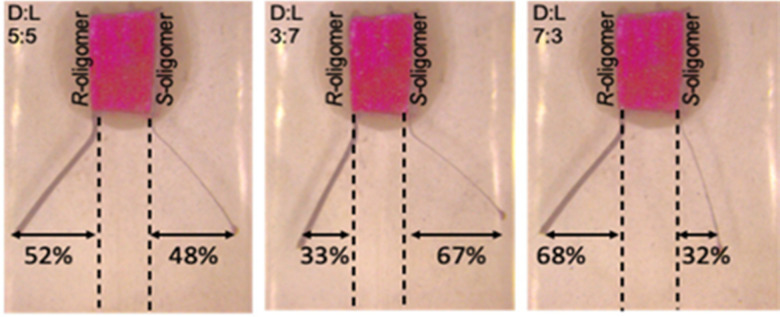
Bipolar electrochemical experiments for the enantiomeric excess determination of solutions with different molar ratios between d- and l-DOPA probes (Copyright © 2021, American Chemical Society).

With a similar philosophy, a double recognition cantilever system was built to simultaneously quantify, through mechanical deflection, both enantiomers of DOPA when they are present in racemic form or as unbalanced mixtures. In this set-up two hybrid actuators functionalized with (*S*)- and (*R*)-oligomers were immobilized onto an inert support on their rough sides (Fig. 7).

When the BPE is exposed to solutions containing different ratios of the analyte, deformations of both cantilevers can be observed. The degree of the deformations was directly correlated to the concentration of each enantiomeric probe in solution. In this set of experiments BT_2_T_4_ was deposited on the smooth side of the Ppy to obtain an outwards bending of both the strips (Fig. 7).^80^

### Chiral cantilevers as chiral valves

**Fig. 8:**
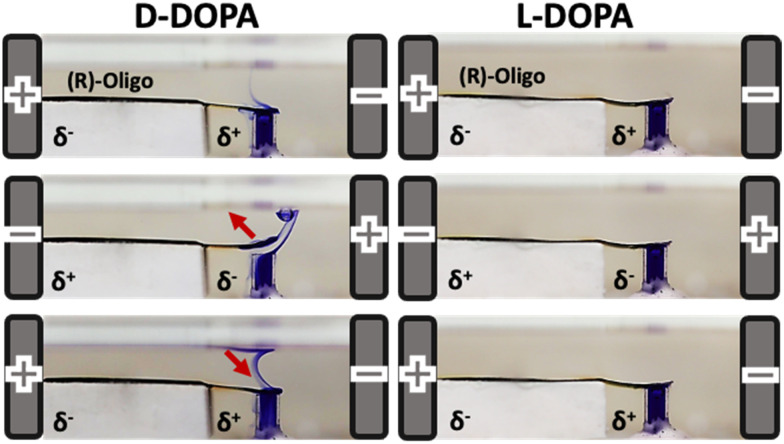
Bipolar enantioselective valve operating with a Ppy actuator functionalized with (*R*)-oligo-BT_2_T_4_ dipped in an aqueous 0.2 M LiClO_4_ solution of 5 mM d-DOPA on the left and 5 mM l-DOPA on the right (Copyright © 2022, John Wiley and Sons).

Wireless enantio-responsive valves triggered by bipolar electrochemistry combined with chiral recognition were also developed. A Ppy actuator functionalized with the enantiomers of an inherently chiral oligomer was used as a bipolar valve to cover a tube loaded with a dye. In the presence of the correspondent chiral analyte, when a proper electric field is applied, the designed actuator has shown a reversible cantilever-type deflection, allowing the release of the dye from the reservoir (Fig. 8). The tube can be opened and closed by simply switching the polarity of the electrochemical system. Furthermore, by using a double enantio-responsive cantilever system, functionalized with (*S*)- and (*R*)-oligomers respectively, it is possible to detect different chiral analytes in a single experiment. Therefore, the device works well even in the presence of chemically different chiral analytes in the same solution. Finally, although the proposed set-up has characteristic dimensions in the millimeter scale, in principle they can be scaled down to smaller dimensions in order to design micro-valves for microfluidics.^81^

## Outlook

While much of the work on hybrid actuators has focused on achiral systems, that need a connection to a power supply with difficulties in scaling down, there is a growing need for methods that involve both chirality and wireless-mode features. A number of studies in the literature have demonstrated that the enantiomers of chiral analytes can be recognized trough electrochemistry on chiral electrode surfaces but in terms of current intensities. The introduction of inherently chiral materials in electrochemical applications allows enantiospecific electrodes that are able to discriminate chiral probes in terms of thermodynamic potential values to be obtained. This is particularly convenient for determinations when both the enantiomeric analytes are present and is even more crucial for applications in bipolar electrochemistry. In such techniques it is necessary to produce a polarization potential difference to activate the redox reactions at the extremities of the bipolar electrode. By functionalizing the BPE with inherently chiral materials, it is possible to selectively oxidise or reduce only one of the two enantiomers of the chiral probe under study. Such a feature is related to the high difference in the oxidation potentials between the two probe enantiomers when tested on these enantiospecific intrinsically chiral surfaces. Bipolar electrochemistry is a wireless technique that can be exploited in different fields, especially when coupled with a complex physico-chemical property such as chirality. The development of electrochemical systems, that throughout a dynamic event, translate chirality from the molecular to the macroscopic scale, is highly desired and a hot topic. This should open up interesting perspectives for the possible use of this approach as an alternative and straightforward tool in the electroanalysis of samples containing chiral molecules. In this frame, the striking enantiodiscrimination ability of inherently chiral compounds has opened new ways for mechanical and optical read-outs.^82–85^ For example, recently a novel autonomous enzyme-based swimmer able to convert chiral information at the molecular level into macroscopic enantioselective trajectories was designed.^84^ With a similar philosophy wireless enantioselective rotors, where the angle of rotation was used as a readout of chiral information, were designed.^85^ Furthermore, enantiorecognition by means of light emitting devices was achieved by coupling inherently chiral compounds and microelectronic objects.^82,83^ All these outstanding approaches allow correlation of the output signal (*i.e.* the dynamic event of a macroscopic object or light emission) with the concentration and chirality of the electroactive enantiomers dissolved in the working solution, even in the case of mixtures containing different ratios of the probe antipodes. In the near future we are planning to build-up chiral micro-cargo-towing reactors that are able to decipher the chirality of probes under study and to cargo-deliver them to the desired destination. These systems will be triggered by three main ingredients; chirality, electric and magnetic fields.

## Author contributions

SA wrote and edited the manuscript.

## Conflicts of interest

There are no conflicts to declare.

## Supplementary Material
